# Dietary intake of young twins: nature or nurture?^1^,^2^,^3^

**DOI:** 10.3945/ajcn.113.065250

**Published:** 2013-09-18

**Authors:** Laura Pimpin, Gina L Ambrosini, Clare H Llewellyn, Laura Johnson, Cornelia HM van Jaarsveld, Susan A Jebb, Jane Wardle

**Affiliations:** 1 From Diet and Population Health, Medical Research Council Human Nutrition Research, Cambridge, United Kingdom (LP, GLA, and SAJ); Health Behaviour Research Centre, the Department of Epidemiology and Public Health, University College London, London, United Kingdom (CHL, CHMvJ, and JW); and the Centre for Exercise, Nutrition, and Health Sciences, School for Policy Studies, University of Bristol, Bristol, United Kingdom (LJ).

## Abstract

**Background**: The early years in life are increasingly recognized as a critical period for the development of diet-related behavioral traits. However, discussions continue on the relative role of genes and the environment in determining dietary intake, particularly in young children for whom detailed dietary information is limited.

**Objectives**: This study tested the hypothesis that diet in early childhood is primarily determined by the environment rather than by genes. A secondary aim was to characterize the early childhood diet.

**Design**: A classic twin design used 3-d dietary data collected at age 21 mo from the Gemini cohort. From the full sample of 2402 families with twins, dietary diaries were available for 1216 twin pairs (384 monozygotic and 832 dizygotic pairs) after exclusions. Intakes of macronutrients, food, and beverages were estimated. Twin analyses quantified the contributions of genetic and environmental factors to population variation in intake.

**Results**: At age 21 mo, children consumed small portions of a wide range of family foods. The shared environment was the predominant determinant, contributing between 66% (95% CI: 52%, 77%; milk-based desserts) and 97% (95% CI: 95%, 98%; juice) of the variation in intake. Genetic factors were estimated to account for between 4% (95% CI: 0%, 10%; savory snacks) and 18% (95% CI: 14%, 23%; bread) of dietary intake variation.

**Conclusion**: Shared environmental influences are the predominant drivers of dietary intake in very young children, indicating the importance of factors such as the home food environment and parental behaviors.

## INTRODUCTION

A large body of research has investigated the early life dietary risk factors for obesity and chronic diseases (1). Information on the drivers and determinants of poor diets at a young age is crucial to identify targets for interventions to improve dietary intake quality and, ultimately, health outcomes. These determinants may have environmental or genetic origins; for instance, the influence of parental dietary intake, which is consistently identified as the strongest correlate of young children's intake (2), can be mediated through both pathways.

Twin data indicate that food neophobia in children is quite strongly heritable at age 9 y, whereas food preferences are no more than moderately heritable in children aged 4–5 and 9 y (3–5), with heritability varying by food type and being stronger for meat and fish than for fruit, vegetables, or desserts in 4- to 5-y-olds (4). There is also evidence that appetitive traits such as satiety responsiveness and enjoyment of food are determined by genetic factors in children to a higher degree than food preferences (6, 7), and the same appetitive traits have been shown to be related to genetic pathways linked to weight gain (8). However, very little research has been undertaken on the influence of genetics and the environment on actual dietary intake in very young children, in part because of the paucity of good-quality dietary data in this age group. One study in 7-y-olds found considerable variability in heritability estimates for 24-h food and beverage intake, ranging from 12% to 79%, which was not consistent between sexes (9).

During early childhood, twins share the same environmental exposures and either 100% (monozygotic twin pairs) or ∼50% (dizygotic twin pairs) of their genetic material. Children's food intake might be expected to be partly genetically determined by drivers such as preferences (10), but at young ages children are largely reliant on their immediate family for food. It was therefore hypothesized that at the complementary feeding stage, children's macronutrient and food intakes would be predominantly driven by environmental rather than genetic factors.

## SUBJECTS AND METHODS

### Study sample

Subjects were participants in the Gemini study, a birth cohort of 2402 pairs of twins recruited by using birth registration data for all twins in England and Wales from March to December 2007. Families who agreed to participate were sent questionnaires and requests for weight and height data and completed a 3-d dietary diary when twins were, on average, 21 mo old (11). Gemini is one of few twin studies to have obtained detailed information on a broad range of early behaviors and traits pertaining to nutrition, growth, and development in very young children. Ethical approval for Gemini was granted by the University College London Committee for the Ethics of Non–National Health Service Human Research, and all aspects of the data collection and storage were in accordance with the standards stipulated by this committee.

### Zygosity

Parents were asked whether their twins were opposite- or same-sex twins. Opposite-sex twins were classified as dizygotic, and parents of same-sex twins completed a validated 20-item zygosity questionnaire (12) at 2 time points [when the twins were, on average, 8.2 mo old (SD: 2.2 mo) and 2.4 y old (SD: 0.25 y)]. In addition, zygosity was established by using DNA in a sample of 311 twin pairs who could not be classified by their questionnaire responses. The questionnaire was 100% accurate for zygosity allocation in a random sample of 81 pairs who were tested for DNA in the Gemini sample (13).

### Dietary data collection

Parents recorded food and drink intake for both children between November 2008 and August 2009, when the twins were, on average, 21 mo old (mean ± SD: 20.8 ± 1.2 mo; range: 17.3–34.2 mo). Detailed instructions were provided to parents and carers on how to accurately estimate and record all food and drinks consumed by each twin for 3 d (any 2 weekdays and 1 weekend day) while in their care (14). Once returned, the diaries were checked, coded, and linked with British food composition tables (15) to provide average daily intakes of energy, macronutrients, and foods by using Diet In Nutrients Out (DINO), an in-house program developed at Medical Research Council Human Nutrition Research (Cambridge) (16). Foods were grouped into 22 categories on the basis of nutrient profiles and a priori knowledge of the main types of food consumed by young children (17).

Of the 2714 diaries submitted (56% of the cohort), those that included only 1 recorded day were excluded because these may not be adequately representative of a habitual diet (*n* = 122), providing data on 2592 children who had complete data for 2 or 3 d (18). Analyses were also restricted to diaries completed within a defined age range (17–28 mo), which excluded 2 diaries from older children. Diaries with >28 d between the first and last day of diary entry were also excluded (*n* = 132) to ensure that the data represented the intake at a given month of age. Finally, diaries from twins with unknown zygosity were excluded (*n* = 26), which left diaries for a total of 2432 twins for analysis (50.6% of the cohort) (**Table 1**). Despite instructions, some included diaries were not completed using a combination of weekdays and weekend day (*n* = 684; 28%); however, these were included, because diet for this age group is unlikely to be greatly biased by the day of the week reported.

**TABLE 1:** Demographic characteristics of the Gemini sample and dietary diary respondents

	Total Gemini sample	Gemini dietary diary respondent sample	*P* for between-group difference
Number of twins enrolled	4804	2432	
Sex [*n* (%)]			
Male	2386 (49.7)	1187 (48.8)	0.729*^1^*
Female	2418 (50.3)	1245 (51.2)	
Age at baseline questionnaire (mo)	7.8 (6.5–9.7)*^2^*	7.6 (6.5–9.4)	<0.001*^3^*
Number of Gemini twin families	2402	1216	
Sex and zygosity of twin pairs [*n* (%)]			
Monozygotic males	352 (14.7)	185 (15.2)	0.068*^1^*
Dizygotic males	409 (17.1)	201 (16.5)	
Monozygotic females	397 (16.5)	199 (16.4)	
Dizygotic females	391 (16.3)	216 (17.8)	
Dizygotic opposite sex	816 (34.0)	415 (34.1)	
Unknown	37 (1.5)	0 (0)	
Ethnic group of mother [*n* (%)]			
White	2231 (92.9)	1155 (95.0)	<0.001*^1^*
Mixed/multiple ethnicities	46 (1.9)	18 (1.5)	
Asian	72 (3.0)	26 (2.1)	
Black/Caribbean	45 (1.9)	14 (1.2)	
Other	6 (0.3)	3 (0.2)	
Unknown	2 (0.1)	0 (0)	
Household socioeconomic status [*n* (%)]			
Low	472 (19.7)	182 (15.0)	<0.001*^1^*
Medium	407 (16.9)	178 (14.6)	
High	1515 (63.1)	853 (70.2)	
Unknown	8 (0.3)	3 (0.3)	
Age of mother at twin birth (y)	33.4 (29.6–36.4)*^4^*	34.1 (31.1–36.9)*^5^*	<0.001*^3^*
BMI of mother at baseline questionnaire (kg/m^2^)	24.1 (21.7–27.5)*^6^*	23.8 (21.5–27.0)*^7^*	<0.001*^3^*
Mother BMI (in kg/m^2^) classification [*n* (%)]			
Underweight (≤18.49)	112 (2.3)	44 (1.8)	<0.001*^1^*
Normal weight (18.5–24.9)	2572 (53.5)	1434 (58.9)	
Overweight (25.0–29.9)	1358 (28.3)	680 (28.0)	
Obese (≥30)	634 (13.2)	238 (9.8)	
Unknown	128 (2.7)	36 (1.5)	

1 Chi-square test for difference between populations.

2 Median; IQR in parentheses (all such values).

3 Mann-Whitney *U* test and for difference between populations.

4 *n* = 4792.

5 *n* = 2430.

6 *n* = 4676.

7 *n* = 2396.

### Dietary variable description

Macronutrient intakes were normally distributed and were therefore described according to absolute mean (±SD) daily intakes in grams per day (**Table 2**) and proportions of total energy intake (**Table 3**). Intakes of most foods were not normally distributed and so were summarized by using median (IQR) daily intakes (g/d) (Table 2). The average daily energy intake (kJ/d) for each food group and the proportion of total daily energy intake (%) from each food group were estimated both for the whole study population and specifically for consumers (Table 3). Data for sauces, condiments and soups, and supplements and nutrition powder food groups are not presented because these were heterogeneous and seldom consumed.

**TABLE 2:** Average daily macronutrient and food group intakes at age 21 mo and intraclass correlation coefficients for consumers by zygosity: Gemini twin cohort*^1^*

			Intraclass correlation (95% CI)*^2^*	
	At least one twin per pair consuming	Intake of consumers	Monozygotic pairs	Dizygotic pairs
	*%*			
Total food weight (g/d)	100	1222 ± 271*^3^*	0.98 (0.98, 0.98)	0.94 (0.94, 0.95)
Total energy intake (kJ/d)	100	4334 ± 783	0.96 (0.95, 0.96)	0.93 (0.92, 0.93)
Total protein intake (g/d)	100	40 ± 9	0.97 (0.96, 0.97)	0.93 (0.93, 0.94)
Total fat intake (g/d)	100	42 ± 10	0.97 (0.96, 0.97)	0.94 (0.93, 0.94)
Total carbohydrate intake (g/d)	100	132 ± 27	0.96 (0.96, 0.97)	0.93 (0.93, 0.94)
Liquids (g/d)				
Milk	98	373 (242–488)*^4^*	0.96 (0.95, 0.97)	0.90 (0.89, 0.91)
Water	98	231 (148–358)	0.98 (0.98, 0.99)	0.91 (0.90, 0.92)
Formula milk	15	275 (196–392)	0.99 (0.98, 0.99)	0.80 (0.73, 0.86)
Breast milk	2	200 (100–300)	0.95 (0.87, 1.00)	0.99 (0.99, 1.00)
Juice	69	57 (23–113)	0.98 (0.98, 0.98)	0.93 (0.92, 0.95)
Other beverages	14	18 (6–84)	0.98 (0.98, 0.99)	0.95 (0.93, 0.97)
Solid foods (g/d)				
Bread	97	36 (25–50)	0.92 (0.91, 0.94)	0.84 (0.82, 0.86)
Dairy	98	72 (52–101)	0.93 (0.92, 0.95)	0.86 (0.85, 0.88)
Vegetables	98	65 (41–93)	0.95 (0.94, 0.96)	0.89 (0.88, 0.90)
Fruit	98	113 (75–157)	0.91 (0.90, 0.93)	0.87 (0.85, 0.88)
Cereal products	99	53 (33–81)	0.94 (0.93, 0.95)	0.88 (0.87, 0.90)
Potato	90	45 (31–63)	0.89 (0.87, 0.91)	0.85 (0.83, 0.87)
Fats and oils	94	8 (5–11)	0.89 (0.87, 0.92)	0.81 (0.78, 0.83)
Milk-based desserts	26	75 (60–105)	0.89 (0.85, 0.93)	0.92 (0.90, 0.94)
Meat and fish	97	44 (30–63)	0.92 (0.90, 0.93)	0.90 (0.89, 0.92)
Sweet cereal-based products	81	23 (14–37)	0.91 (0.89, 0.93)	0.86 (0.85, 0.88)
Commercial infant foods	55	50 (14–100)	0.96 (0.95, 0.97)	0.96 (0.95, 0.96)
Savory snacks	65	11 (7–17)	0.94 (0.93, 0.95)	0.88 (0.86, 0.90)
Added sugars and confectionery	73	12 (6–19)	0.98 (0.97, 0.98)	0.91 (0.89, 0.92)
Egg	44	42 (16–60)	0.95 (0.93, 0.96)	0.88 (0.85, 0.90)

1 Milk: skimmed, semiskimmed and whole cow milk, other animal-based milk, plant-based milk, milk-based drinks; formula milk: all formula milks; juice: fruit-based drinks, baby/infant processed drinks; other beverages: carbonated soft drinks, powdered beverages, coffee, tea, alcohol from composite dishes; bread: white, whole-grain, brown, seeded bread, wheat-germ, other breads, crisp breads; dairy: cream, *fromage frais*, cheese, yogurt, liquid yogurt, ice cream, dairy desserts; vegetables: tomatoes; cruciferous vegetables; yellow, red, and dark green leafy vegetables; other vegetables; pulses; lentils; baked beans; fruit: fresh, dried, canned, and cooked fruit; cereal products: pizza, pasta and pasta-based meals, rice and rice-based meals, other cereals and other cereal-based meals, oat-based cereals, other breakfast cereals; potato: potatoes, potato products; fats and oils: butter, oils, animal-based fats, plant-based fats; meat and fish: white fish; oily fish; shellfish; beef, veal, beef- and veal-based dishes; lamb and lamb-based meals; pork and pork-based meals; other red meat; venison; chicken, turkey, and venison-, chicken-, or turkey-based meals; other game birds; bacon and ham; processed pies; other processed meats; sausages, burgers, and kebabs; liver and liver-based meals; other offal and offal-based meals; sweet cereal-based products: biscuits, pastries, buns, pies, cereal-based desserts (not milk), cereal bars; commercial infant foods: ready meals, manufactured fruit-only purees, biscuits, dried cereals; savory snacks: nuts, seeds, potato-based snacks, cereal-based snacks, vegetable-based snacks, savory biscuits, crackers; added sugars and confectionery: jam; marmalade; chutney; pickles; pure sugars; other sugars including syrups, honey, chocolate-based products, sugar-based products, and sorbets; egg: egg and egg-based meals.

2 Only twin pairs where one or both twins was a consumer are included.

3 Mean ± SD (all such values).

4 Median; IQR in parentheses (all such values).

**TABLE 3:** Energy from macronutrient and food group intakes at 21 mo of age: the Gemini twin cohort (whole study population and consumers)*^1^*

	All		Consumers only		
	Daily energy intake	Proportion of total daily energy intake	Consumers	Daily energy intake	Proportion of total daily energy intake
		*%*	*%*		*%*
Macronutrients (%)					
Proportion of energy from protein	16 ± 2*^2^*	N/A	N/A	N/A	N/A
Proportion of energy from fat	36 ± 5	N/A	N/A	N/A	N/A
Proportion of energy from carbohydrate	49 ± 6	N/A	N/A	N/A	N/A
Liquids (kJ/d)					
Milk	981 (599–1306)*^3^*	23 (14–30)	98	995 (647–1318)	23 (15–30)
Water	0 (0– 0)	0 (0–0)	98	0 (0–0)	0 (0–0)
Formula milk	0 (0–0)	0 (0–0)	15	804 (567–1140)	19 (13–28)
Breast milk	0 (0–0)	0 (0–0)	2	578 (289–867)	11 (7–18)
Juice	11 (0–93)	0 (0–2)	69	51 (10–147)	1 (0–4)
Other beverages	0 (0–0)	0 (0–0)	14	5 (2–48)	0 (0–1)
Solid foods (kJ/d)					
Bread	378 (254–529)	9 (6–12)	97	384 (269–535)	9 (6–12)
Dairy	422 (294–604)	10 (7–14)	98	426 (309–609)	10 (7–14)
Vegetables	116 (64–193)	3 (2–5)	98	118 (69–196)	3 (2–5)
Fruit	363 (228–497)	8 (5–11)	98	367 (238–503)	9 (6–12)
Cereal products	474 (326–665)	10 (11–19)	99	475 (332–668)	11 (8–16)
Potato	187 (98–283)	4 (2–7)	90	206 (132–299)	5 (3–7)
Fats and oils	186 (104–282)	4 (3–6)	94	197 (120–292)	5 (3–7)
Milk-based desserts	0 (0–33)	0 (0–1)	26	304 (228–457)	7 (5–10)
Meat and fish	341 (214–511)	8 (5–12)	97	352 (231–520)	8 (6–12)
Sweet cereal-based products	318 (135–514)	7 (3–12)	81	381 (253–583)	9 (6–13)
Commercial infant foods	86 (0–309)	2 (0–7)	55	289 (168–478)	7 (4–11)
Savory snacks	130 (0–274)	3 (0–6)	65	231 (139–365)	5 (3–8)
Added sugars and confectionery	98 (0–231)	2 (0–5)	73	165 (84–288)	4 (2–6)
Egg	0 (0–205)	0 (0–5)	44	299 (109–410)	6 (3–10)

1 *See* Table 2 footnote 1 for definitions of food groups. N/A, not applicable.

2 Mean ± SD (all such values).

3 Median; IQR in parentheses (all such values).

Demographic characteristics including the child's sex, the mother's ethnic origin, household socioeconomic status [SES; defined by using the National Statistics Socioeconomic Class index on the basis of occupation (19)], age of the mother at the twins’ birth, and BMI of the mother were derived from questionnaires at baseline when the twins were, on average, 8.2 ± 2.2 mo old and summarized by using means ± SDs. Maternal BMI (in kg/m^2^) was categorized as underweight (≤18.49), normal weight (18.5–24.9), overweight (25–29.9), or obese (≥30) (20). The exact age of the twins at the time of completion of the dietary diary was summarized by using medians (IQRs).

### Statistical analyses

#### Sample representativeness

Differences in demographic characteristics between the dietary diary responders (*n* = 1216 pairs of twins; Table 1) and the total Gemini sample (*n* = 2402 pairs) were examined to assess the representativeness of the dietary diary sample.

#### Heritability analyses

The main principle of the use of twins to model heritability and environmental effects relies on comparing the degree of concordance between monozygotic and dizygotic twin pairs for a given trait. Greater similarity between monozygotic pairs compared with dizygotic pairs indicates greater genetic contribution to the population variation for that trait. Greater twin similarity than would be predicted on the basis of the estimated heritability is an estimate of the influence of the shared environment.

Analyses were performed on age- and sex-standardized residual scores of average daily energy (kJ/d), macronutrient (g/d), and food (g/d) intakes (Table 2) to account for the exact correlation between age and sex of twins within same-sex pairs, because these can inflate the shared environment effect (21). It was not possible to normalize the distribution of some food groups by using transformation methods, so these were dichotomized by splitting along the median of the residual intake scores for each food group (Table 2); these food groups included eggs, savory snacks, other beverages, juice, meat and fish, water, commercial infant foods, formula milk, and added sugars and confectionery foods. Pairs in which neither twin consumed a food were excluded from analyses of that particular food group, because it was not possible to ascertain whether both twins were offered and both refused to consume this food or whether they were not offered the food. This was done to avoid inflation of the shared-environment effect, because concordance for zero intake due to not being offered the food does not necessarily reflect concordance in intakes but contributes to the within-pair correlation as if both twins consumed the same amount of food.

As a first indicator of genetic and environmental influence, intraclass correlations were calculated for each dietary component within twin pairs, whereas tetrachoric correlations were calculated for dichotomized variables (Table 2) by using Stata, release 12 (StataCorp LP). Standard ACE structural equation modeling was then used to estimate the relative proportion of overall variance in macronutrient, energy, and food intakes attributable to additive genetic effects (A), shared environmental influences (C), or unique environmental influences and measurement error (E) (22). Additional fitting of the structural equation models used Mx Maximum Likelihood Structural Equation Modeling software (version 32; Virginia Commonwealth University). This calculated the 95% CI for each variable estimate, tested the fit and the assumptions of the complete ACE model, and tested the goodness-of-fit of nested submodels that sequentially drop the A parameter, the C parameter, or both the A and C parameters (23). The Bayesian information criterion was used to compare the fit of the models because it takes into account the size of the sample, which varies between dietary intake variables depending on the proportion of twins consuming each food. When comparing nested submodels with the complete ACE model, the model with the lowest Bayesian information criterion indicates the best-fitting model (24). The ACE model was selected over the ADE model [which models nonadditive genetic effects (D)], because dizygotic correlations were more than half the monozygotic correlations for the majority of foods, the hallmark of shared environmental effects (C).

## RESULTS

### Responders

Dietary diaries were returned by children with a mean (±SD) age of 20.8 ± 1.2 mo at diary completion. Responders were representative of the whole Gemini sample in terms of sex and zygosity; however, there was a slight overrepresentation of children whose mothers were older, white, of higher SES, and less likely to be obese (*P* < 0.001) (Table 1).

### Dietary intake

The postweaning diet of the children at ∼21 mo of age consisted predominantly of small portions of common family foods. At least one twin in ˃97% of pairs in the sample consumed common family foods such as cereal products, dairy, bread, meat and fish, fruit, and vegetables on at least one occasion during data collection (Table 2), with a low consumption of commercial infant foods (7% of total daily energy in consumers) (Table 3).

More than 60% of twins consumed juice (including 100% juice fruit-based drinks and processed fruit drinks designed for infants), potatoes and potato products, sweet cereal-based products, fats and oils, added sugars and confectionery, and savory snacks. Less commonly consumed foods included other beverages such as tea, coffee, sugar-sweetened drinks, formula milk, breast milk, milk-based desserts, commercial infant foods, and egg and egg-based dishes (Table 2). The most commonly consumed foods contributed the greatest proportion of average total daily energy intake in the whole sample (consumers and nonconsumers), except for vegetables (excluding potatoes), which contributed only 3% of the population's average daily energy intake because of their low energy density (Table 3).

In consumers only, which gave a restricted sample, the most widely consumed foods with regard to contribution to daily energy intake were milk (23% of total daily energy intake), other dairy products (10%), and cereals (11%) (Table 3). Some foods, particularly commercial infant foods, eggs and egg-based meals and milk-based desserts, had a low proportion of consumers but contributed a larger proportion of daily energy among consumers than for the whole sample (7% compared with 2%, 6% compared with 0%, and 7% compared with 0%, respectively).

A minority of children (15%) were still consuming formula milk, and 2% consumed breast milk (Table 2). Most (67%) of the formula milk consumed was age appropriate (follow-on/toddler milk); however, 19% of children still consumed infant formula. Although formula and breast milk contributed <1% of the overall sample's total average daily energy intake, they provided an important part of total daily energy intake for consumers (19% and 11%, respectively) (Table 3).

### Genetic and environmental influences

#### Within-pair correlations

Within-pair correlations for intakes of most macronutrients and foods were high (intraclass correlation coefficient >0.8), suggesting that, within families, children had very similar diets (Table 2). Nonetheless, intakes among monozygotic pairs showed slightly stronger correlations than for dizygotic pairs for most foods, with the exception of breast milk, commercial infant foods, and milk-based desserts. Although CIs did not overlap for dizygotic and monozygotic pairs for the majority of foods, the magnitude of these differences was small, suggesting a very modest role for genetics in determining dietary intake (Table 2).

#### ACE covariance modeling

Genetic and environmental effects were also estimated by using covariance modeling. The genetic component (A) was comparatively small but significant for the majority of intake measures examined; the estimates from full ACE models are therefore shown in **Table 4**. The CE model, excluding the genetic component, showed the best fit for 10 of the food groups (formula milk, juice, other beverages, milk-based desserts, meat and fish, sweet cereal-based products, commercial infant foods, savory snacks, added sugars and confectionery, and eggs); estimates from this submodel are also shown for these food groups in Table 4. It was not possible to perform ACE analyses on breast-milk intake, because only 22 pairs of twins were consumers of breast milk.

**TABLE 4:** Relative contributions (95% CIs) of genetic and environmental factors to variation in energy, macronutrient, and food group intake*^1^*

	Additive genetic effect (A)	Shared environmental effect (C)	Nonshared environmental effect (E)
Macronutrients*^2^*			
Total energy, kJ/d	0.12 (0.08, 0.15)	0.80 (0.77, 0.83)	0.09 (0.07, 0.10)
Total protein, g/d	0.12 (0.09, 0.16)	0.81 (0.78, 0.84)	0.07 (0.06, 0.08)
Total fat, g/d	0.11 (0.08, 0.14)	0.82 (0.79, 0.85)	0.07 (0.06, 0.08)
Total carbohydrate, g/d	0.09 (0.05, 0.12)	0.83 (0.80, 0.86)	0.08 (0.07, 0.10)
Proportion of energy from protein	0.08 (0.06, 0.10)	0.87 (0.85, 0.89)	0.05 (0.04, 0.06)
Proportion of energy from fat	0.10 (0.07, 0.12)	0.86 (0.83, 0.88)	0.05 (0.04, 0.06)
Proportion of energy from carbohydrate	0.09 (0.07, 0.12)	0.86 (0.84, 0.88)	0.05 (0.04, 0.06)
Liquids, g/d			
Milk*^2^*	0.08 (0.05, 0.11)	0.86 (0.83, 0.88)	0.06 (0.05, 0.07)
Water*^2^*,*^3^*	0.07 (0.02, 0.12)	0.91 (0.86, 0.95)	0.02 (0.01, 0.04)
Formula*^3^*,*^4^*			
ACE	0.05 (0.00, 0.32)	0.88 (0.64, 0.96)	0.07 (0.01, 0.18)
CE	—	0.91 (0.82, 0.96)	0.09 (0.04, 0.17)
Juice*^3^*,*^4^*			
ACE	0.01 (0.00, 0.07)	0.96 (0.91, 0.98)	0.04 (0.03, 0.05)
CE	—	0.97 (0.95, 0.98)	0.03 (0.02, 0.05)
Other beverages*^3^*,*^4^*			
ACE	0.05 (0.00, 0.16)	0.95 (0.83, 0.99)	0.00 (0.00, 0.03)
CE	—	0.99 (0.96, 1.00)	0.01 (0.00, 0.04)
Solid foods, g/d			
Bread*^2^*	0.18 (0.14, 0.23)	0.73 (0.69, 0.77)	0.09 (0.07, 0.10)
Dairy*^2^*	0.17 (0.14, 0.21)	0.76 (0.72, 0.80)	0.07 (0.06, 0.08)
Vegetables*^2^*	0.15 (0.12, 0.18)	0.81 (0.78, 0.83)	0.05 (0.04, 0.05)
Fruit*^2^*	0.10 (0.06, 0.13)	0.82 (0.79, 0.85)	0.09 (0.07, 0.10)
Cereal products*^2^*	0.09 (0.06, 0.12)	0.84 (0.81, 0.86)	0.08 (0.06, 0.09)
Potato*^2^*	0.09 (0.04, 0.15)	0.78 (0.74, 0.82)	0.13 (0.11; 0.15)
Fats and oils*^2^*	0.05 (0.00, 0.09)	0.84 (0.80, 0.87)	0.12 (0.10; 0.14)
Milk-based desserts*^4^*			
ACE	0.15 (0.00, 0.30)	0.66 (0.52, 0.77)	0.19 (0.14, 0.26)
CE	—	0.76 (0.71, 0.80)	0.24 (0.20, 0.29)
Meat and fish*^3^*,*^4^*			
ACE	0.09 (0.00, 0.18)	0.86 (0.77, 0.93)	0.06 (0.03, 0.10)
CE	—	0.91 (0.89, 0.94)	0.09 (0.06, 0.11)
Sweet cereal-based products*^4^*			
ACE	0.05 (0.00, 0.10)	0.84 (0.80, 0.87)	0.11 (0.09, 0.14)
CE	—	0.87 (0.85, 0.88)	0.13 (0.12, 0.15)
Commercial infant foods*^3^*,*^4^*			
ACE	0.05 (0.00, 0.11)	0.94 (0.88, 0.98)	0.01 (0.00, 0.03)
CE	—	0.97 (0.96, 0.99)	0.03 (0.02, 0.04)
Savory snacks*^3^*,*^4^*			
ACE	0.04 (0.00, 0.10)	0.94 (0.88, 0.97)	0.03 (0.01, 0.06)
CE	—	0.96 (0.94, 0.97)	0.04 (0.03, 0.06)
Added sugars and confectionery*^3^*,*^4^*			
ACE	0.05 (0.00, 0.13)	0.91 (0.84, 0.96)	0.04 (0.02, 0.08)
CE	—	0.94 (0.92, 0.96)	0.06 (0.04, 0.08)
Egg*^3^*,*^4^*			
ACE	0.06 (0.00, 0.15)	0.91 (0.82, 0.97)	0.03 (0.01, 0.07)
CE	—	0.95 (0.92, 0.97)	0.05 (0.03, 0.08)

1 *See* Table 2 footnote 1 for definitions of food groups. Dashes indicate that the parameter was not included in the model.

2 The best-fitting model (ACE) is presented (*see* Supplemental Table 1 under “Supplemental data” in the online issue for details of saturated and nested submodels).

3 Variables not normally distributed were dichotomized by splitting along the median intake.

4 The ACE model and best-fitting model (CE) are presented.

For energy and macronutrient intakes, the proportion of variance explained by genetic effects ranged from 8% (95% CI: 6%, 10%) for percentage of total energy from protein to 12% (95% CI: 8%, 15%) for total energy intake (kJ/d) and 12% (95% CI: 9%, 16%) for protein intake (g/d). The proportion of variance explained by shared environmental effects ranged from 80% (95% CI: 77%, 83%; energy) to 87% (95% CI: 85%, 89%; percentage of total energy from protein) (Table 4). Estimated CIs and model fit statistics are shown in Table 4 and in Supplemental Table 1 (under “Supplemental data” in the online issue).

The results for food groups were of a similar magnitude, although more varied. For food groups in which the full ACE model was the best fitting, the proportion of variance in intake explained by genetic factors was low, ranging from 5% (95% CI: 0%, 10%) in sweet-cereal products and fats and oils to 18% (95% CI: 14%, 23%) for bread. On the other hand, shared environmental effects explained 66% (95% CI: 52%, 77%) of variance for milk-based desserts to 91% for added sugars and confectionery (95% CI: 88%, 97%) and water (95% CI: 86%, 95%) (Table 4).

Beverages and food group intakes that were dichotomized for analysis because of their highly skewed distributions showed a wider range of A, C, and E estimates. However, for the majority, estimates for the shared-environment components were large (>66%) and estimates for the genetic component were nonsignificant.

Estimates for the E component, which represents measurement error as well as nonshared environmental exposures specific to the individual and differ between twins within a pair, were low. They ranged between 1% (95% CI: 0%, 0.04%) for the best-fitting CE model for other beverages to 24% (95% CI: 20%, 29%) for the CE model for milk-based desserts; however, the vast majority of dietary variables varied between 3% and 9%, indicating the presence of measurement error as well as a limited role of individual-level environmental factors in determining variability in dietary intake.

## DISCUSSION

The main aim of this study was to quantify the contribution of genes and environment to the variation in energy, macronutrient, food, and beverage intakes in young children by using data from the Gemini twin birth cohort.

The results from this analysis show that shared environmental influences are the primary determinants of dietary intake in 21-mo-olds, with genetic effects explaining small, although significant, additional variation in dietary intake. As such, elements of the immediate environment common to both twins, such as parental intake and feeding styles, the availability of food in the home, or the foods offered to the children are likely to be the principal drivers of dietary intake at this age (2), although these may also include broader determinants such as SES and ethnicity. Parents and carers have the primary responsibility in providing food, and children at this age can probably only partially express their inherent preferences with regard to food type, although they can determine how much they eat of the foods they are offered. This highlights the importance of the home food environment and child feeding practices in determining healthy dietary habits in young children.

Placing these findings within the context of current knowledge is challenging, because little research has been undertaken to quantify the determinants of very young children's dietary intake. Estimates of heritability in this study were similar to those found in a study in 396 twins aged 7 y (9), in whom the highest heritability estimate was 18%. The best-fitting models in the current study tended to be those with a minimal contribution of genetics, which is comparable to Faith et al's (9) study, in which many of the dietary food and beverage intakes showed the best fits for the CE models, which dropped the heritability component entirely. However, heritability estimates varied considerably by food group and sex for the 7-y-olds studied: whereas shared environment was the principal driver of intake in girls, genetic predisposition played a more important role in boys. The magnitude of the significant heritability components for both sexes in the study in 7-y-olds was similar to those reported in adult studies (25), which have shown dietary traits such as total energy consumption, specific dietary pattern, and food or individual nutrient intakes to be moderately heritable; most estimated that genetic factors explained 20–40% of the overall variation in dietary traits (26–29). Genomewide association studies in adults have shown associations between BMI or weight and common genetic variants involved in eating behaviors, including satiety regulation and dietary intake [eg, fat mass and obesity associated (*FTO*) and melanocortin 4 receptor (*MC4R*)] (30, 31). This indicates some progress in identifying the genomic basis for genetic influence on diet and, by extension, body size.

Longitudinal studies have reported that the strength of the associations between certain genotypes and phenotypes increases during childhood and adolescence (32). This could mean that genetic effects on BMI-related dietary intakes are not fully expressed early in life and may become stronger as children become more independent.

The strong shared-environment effect observed in this analysis may include positive passive gene-environment correlations insofar as parents may give their children what they themselves prefer to eat. This inflates the shared-environment variable because it affects food intake of both twins, regardless of zygosity. The exclusion of pairs in cases in which both members were nonconsumers is a limitation, but it has the effect of lowering the estimates of the shared environmental effect, because it removes twins who are perfectly correlated for their lack of consumption of a food, regardless of zygosity.

Although the association between early food intake and the development of obesity and associated chronic morbidities has been established, there is still little research into the determinants of food intake in very young children. Parental intake and availability in the home have been associated cross-sectionally with fruit and vegetable intake in preschool children (33). At the weaning stage (on average, 14 mo of age), paternal education, SES, parity, and maternal BMI were associated with increased likelihood of adherence to an unhealthy “Western” diet (34). Further research is required to identify modifiable determinants of dietary intake to help develop interventions aimed at improving very young children's diets.

The secondary aim of this study was to characterize diet at a young age by using the twin data because these data had a large amount of detailed dietary information. The data showed that children at ∼21 mo of age were consuming small portions of general family foods. Food diaries have been shown to be accurate for assessing total energy intake in preschoolers when compared with weighed food records and doubly labeled water estimates of energy expenditure (35–37) and to avoid the recall bias inherent in 24-h recall and food-frequency questionnaires. It was not possible to assess the extent of misreporting in this dietary data sample, but the prevalence of misreporters has been found to be smaller in younger populations and is more likely to represent overreporting than underreporting (38). This could potentially lead to an overestimate of the genetic element, because misreporting is related to children's BMI (38) and BMI is strongly genetically influenced (39), although again this is unlikely at this young age.

The results for the few food group intakes that were dichotomized before the heritability analysis, such as for infant formula milk and fruit juice, should not be overinterpreted because there are several indications that they might be an artifact: small numbers of pairs with at least one consumer for formula milk (15%) and other beverages (13%) made their distributions extremely positively skewed, and the reduced variation in the data due to dichotomizing these variables compromises power and accuracy of the parameters, therefore limiting interpretation.

The error involved in measuring any trait or behavior is modeled as part of the E parameter (unique environment component), because this error is assumed to be random (and so individual specific). However, the dietary intakes collected in young twins come from a parent's report of both children's intakes, which may lead to correlated measurement error. The extent of measurement error related to parental reporting is likely to be the same for both twins within a pair and will therefore be modeled as part of the C (shared environmental) component, which may lead to overinflation of this component in the model.

The misclassification of zygosity was minimized by using questionnaires that were validated with the use of DNA data from a subsample of participants. The use of diaries reduced the risk of recall bias because diaries are completed prospectively. Although no dietary assessment method is error free, any residual error in the dietary diaries is unlikely to differ between mothers of monozygotic and dizygotic twins and would therefore be captured under either the C or E component, if the error is respectively correlated between twins or is random, thereby reducing heritability. Age and sex effects, known to be important confounders of children's intakes, were adjusted for in the analysis, which provided fit statistics and CIs to accurately estimate the significance of our findings.

There were some differences between mothers who provided twin dietary data for this analysis and those who did not. Respondents were older, more likely to be white and of higher SES, and less likely to be obese, but the magnitude of differences was small and is unlikely to greatly affect the generalizability to the rest of the cohort or the twin analyses. Generalizing our results to all children is limited by the fact that twins are often small for gestational age and born prematurely. Their lives are also different, because they grow up with a person of the exact same age and are often treated as a “pair” (40). However, comparison with recent data from the Diet and Nutrition Survey of Infants and Young Children for infants aged 4–18 mo (17) and the National Diet and Nutrition Survey for 1.5- to 3-y-olds (41) indicates that the diet of the Gemini twins is broadly similar to the general population from which they were sampled.

In conclusion, environmental effects are substantially stronger influences on the dietary intakes of young children than are genetic factors, and these are mostly shared environmental effects. This highlights the importance of the home food environment and child feeding practices in determining healthy dietary habits in young children. Parents and carers are important targets for education and interventions to improve the diets of toddlers.

## Supplementary Material

Supplemental data
