# Supplementary material for: Dietary intake of young twins: nature or nurture?1
Source: Am J Clin Nutr. 2013 Sep 18;98(5):1326–34. doi: 10.3945/ajcn.113.065250 (PMC3798084; doi:10.3945/ajcn.113.065250)
Supplement: Supplemental data [file supp_98_5_1326__index.html]

Dietary intake of young twins: nature or nurture? — Supplemental data 

# Dietary intake of young twins: nature or nurture?

## Supplemental data

**Files in this Data Supplement:**

- Supplemental data - Table 1
