# Supplementary material for: Dietary intake of young twins: nature or nurture?1
Source: Am J Clin Nutr. 2013 Sep 18;98(5):1326–34. doi: 10.3945/ajcn.113.065250 (PMC3798084; doi:10.3945/ajcn.113.065250)
Supplement: Supplemental data [file 113.065250_ajcn065250SupplementaryData1.doc]

For supplemental online repository

Supplemental Table 1 -Relative contributions of genetic and environmental factors to variation in energy, macronutrient and food group intake, nested sub models and fit statistics

| **Dietary intake variable and model** | **Additive genetic effect (A)** | **Shared environmental effect (C)** | **Non shared environmental effect and measurement error (E)** | **BIC** | **Δ in BIC** | **p-value** |
| --- | --- | --- | --- | --- | --- | --- |
| **Energy (kJ)** |  |  |  |  |  |  |
| Saturated model | - | - | - | -6101.98 |  |  |
| ACE* | 0.12 (0.08; 0.15) | 0.80 (0.77;0.83) | 0.09 (0.07; 0.10) | -6118.34 | -16.363 | 0.732 |
| CE | - | 0.88 (0.87; 0.89) | 0.12 (0.11 ; 0.14) | -6105.18 | 13.159 | <0.001 |
| AE | 0.91 (0.90; 0.92) | - | 0.09 (0.08; 0.10) | -5879.84 | 238.504 | <0.001 |
| E | - | - | 1.00 (1.00; 1.00) | -5222.91 | 895.437 | <0.001 |
| **Protein (g/d)** |  |  |  |  |  |  |
| Saturated model |  |  |  | -6178.99 |  |  |
| ACE* | 0.12 (0.09; 0.16) | 0.81 (0.78; 0.84) | 0.07 (0.06; 0.08) | -6195.78 | -16.791 | 0.858 |
| CE | - | 0.89 (0.88; 0.90) | 0.11 (0.10; 0.12) | -6175.05 | 20.735 | <0.001 |
| AE | 0.93 (0.92; 0.94) | - | 0.07 (0.06; 0.08) | -5931.73 | 264.055 | <0.001 |
| E | - | - | 1.00 (1.00; 1.00) | -5224.36 | 971.425 | <0.001 |
| **Fat (g/d)** |  |  |  |  |  |  |
| Saturated model |  |  |  | -6214.32 |  |  |
| ACE* | 0.11 (0.08; 0.14) | 0.82 (0.79; 0.85) | 0.07 (0.06; 0.08) | -6229.71 | -15.396 | 0.451 |
| CE | - | 0.89 (0.88; 0.90) | 0.11 ( 0.10; 0.12) | -6212.29 | 17.423 | <0.001 |
| AE | 0.93 (0.92; 0.94) | - | 0.07 (0.06; 0.08) | -5954.32 | 275.392 | <0.001 |
| E | - | - | 1.00 (1.00; 1.00) | -5241.41 | 988.309 | <0.001 |
| **CHO (g/d)** |  |  |  |  |  |  |
| Saturated model |  |  |  | -6134.96 |  |  |
| ACE* | 0.09 (0.05; 0.12) | 0.83 (0.80; 0.86) | 0.08 (0.07; 0.10) | -6149.57 | -14.605 | 0.278 |
| CE | - | 0.89 (0.88; 0.90) | 0.11 (0.10; 0.12) | -6141.64 | 7.93 | <0.001 |
| AE | 0.91 (0.90; 0.93) | - | 0.09 (0.08; 0.10) | -5878.14 | 271.428 | <0.001 |
| E | - | - | 1.00 (1.00; 1.00) | -5199.63 | 949.941 | <0.001 |
| **%E from protein** |  |  |  |  |  |  |
| Saturated model |  |  |  | -6340.26 |  |  |
| ACE* | 0.08 (0.06; 0.10) | 0.87 (0.85; 0.89) | 0.05 (0.04; 0.06) | -6356.39 | -16.134 | 0.662 |
| CE | - | 0.92 (0.91; 0.93) | 0.08 (0.07; 0.09) | -6338.82 | 17.568 | <0.001 |
| AE | 0.94 (0.94; 0.95) | - | 0.06 (0.05; 0.06) | -5986.27 | 370.119 | <0.001 |
| E | - | - | 1.00 (1.00; 1.00) | -5183.09 | 1173.3 | <0.001 |
| **%E from fat** |  |  |  |  |  |  |
| Saturated model |  |  |  | -6363.01 |  |  |
| ACE* | 0.10 (0.07; 0.12) | 0.86 (0.83; 0.88) | 0.05 (0.04; 0.06) | -6368.95 | -5.939 | <0.001 |
| CE | - | 0.92 (0.91; 0.93) | 0.08 (0.07; 0.09) | -6344.12 | 24.832 | <0.001 |
| AE | 0.95 (0.94; 0.95) | - | 0.06 (0.05; 0.06) | -6030.81 | 338.136 | <0.001 |
| E | - | - | 1.00 (1.00; 1.00) | -5218.36 | 1150.59 | <0.001 |
| **% from carbohydrates** |  |  |  |  |  |  |
| Saturated model |  |  |  | -6374.27 |  |  |
| ACE* | 0.09 (0.07; 0.12) | 0.86 (0.84; 0.88) | 0.05 (0.04; 0.06) | -6385.19 | -10.919 | 0.018 |
| CE | - | 0.92 (0.91; 0.93) | 0.08 (0.07; 0.09) | -6362.34 | 22.85 | <0.001 |
| AE | 0.95 (0.94; 0.96) | - | 0.05 (0.05; 0.06) | -6030.01 | 355.174 | <0.001 |
| E | - | - | 1.00 (1.00; 1.00) | -5218.819 | 1166.37 | <0.001 |
| **Liquids (g/d)** |  |  |  |  |  |  |
| **Milk (g/d)** |  |  |  |  |  |  |
| Saturated model |  |  |  | -6168.876 |  |  |
| ACE* | 0.08 (0.05; 0.11) | 0.86 (0.83; 0.88) | 0.06 (0.05; 0.07) | -6185.313 | -16.437 | 0.771 |
| CE | - | 0.91 (0.90; 0.92) | 0.09 (0.08; 0.10) | -6174.913 | 10.4 | <0.001 |
| AE | 0.93 (0.92; 0.94) |  | 0.07 (0.06; 0.08) | -5852.622 | 332.691 | <0.001 |
| E | - | - | 1.00 (1.00; 1.00) | -5126.993 | 1058.32 | <0.001 |
| **Water ($) (g/d)** |  |  |  |  |  |  |
| Saturated model |  |  |  | -7414.534 |  |  |
| ACE* | 0.07 (0.02;0.12) | 0.91 (0.86;0.95) | 0.02 (0.01;0.04) | -7421.24 | -6.706 |  |
| CE | - | 0.96 (0.94;0.97) | 0.04 (0.03;0.06) | -7421.489 | -0.249 | 0.01 |
| AE | 0.99 (0.98;0.99) | - | 0.01 (0.01;0.02) | -7302.727 | 118.513 | <0.001 |
| E | - | - | 1.00 (1.00; 1.00) | -6949.605 | 471.635 | <0.001 |
| **Formula (g/d) ($)** |  |  |  |  |  |  |
| Saturated model |  |  |  | -311.079 |  |  |
| ACE | 0.05 (0;0.32) | 0.88 (0.64;0.96) | 0.34 (0.27; 0.43) | -313.95 | -2.871 |  |
| CE* | - | 0.91 (0.82;0.96) | 0.34 (0.26; 0.43) | -712.752 | -398.802 | 0.734 |
| AE | 0.96 (0.87;0.99) | - | 0.38 (0.29; 0.50) | -699.602 | -385.652 | <0.001 |
| E | - | - | 1.00 (1.00; 1.00) | -663.455 | -349.505 | <0.001 |
| **Juice ($) (g/d)** |  |  |  |  |  |  |
| Saturated model |  |  |  | -4767.402 |  |  |
| ACE | 0.01 (0;0.07) | 0.96 (0.91;0.98) | 0.04 (0.03; 0.05) | -4774.182 | -6.78 |  |
| CE* | - | 0.97 (0.95;0.98) | 0.06 (0.05; 0.06) | -4777.507 | -3.325 | 0.784 |
| AE | 0.98 (0.96;0.99) | - | 0.04 (0.04; 0.05) | -4676.35 | 97.832 | <0.001 |
| E | - | - | 1.00 (1.00; 1.00) | -4443.809 | 330.373 | <0.001 |
| **Other beverages ($)** |  |  |  |  |  |  |
| Saturated model |  |  |  | -685.677 |  |  |
| ACE | 0.05 (0;0.16) | 0.95 (0.83; 0.99) | 0.00 (0.00; 0.03) | -692.175 | -6.498 |  |
| CE* | - | 0.99 (0.96; 1.00) | 0.01 (0.00; 0.04) | -693.434 | -1.259 | 0.107 |
| AE | 1 (0.98;1) | - | 0.00 (0.00; 0.02) | -673.22 | 18.955 | <0.001 |
| E | - | - | 1.00 (1.00; 1.00) | -615.915 | 76.26 | <0.001 |
| **Solids (g/d)** |  |  |  |  |  |  |
| **Bread (g/d)** |  |  |  |  |  |  |
| Saturated model |  |  |  | -5911.17 |  |  |
| ACE* | 0.18 (0.14; 0.23) | 0.73 (0.69; 0.77) | 0.09 (0.07; 0.10) | -5923.672 | -12.502 |  |
| CE | - | 0.85 (0.84; 0.87) | 0.15 (0.13; 0.16) | -5896.595 | 27.077 | <0.001 |
| AE | 0.91 (0.90; 0.92) | - | 0.09 (0.08; 0.10) | -5753.287 | 170.385 | <0.001 |
| E | - | - | 1.00 (1.00; 1.00) | -5133.375 | 790.297 | <0.001 |
| **Dairy (g/d)** |  |  |  |  |  |  |
| Saturated model |  |  |  | -5986.297 |  |  |
| ACE* | 0.17 (0.14; 0.21) | 0.76 (0.72; 0.80) | 0.07 (0.06; 0.08) | -6001.226 | -14.929 | inc |
| CE | - | 0.87 (0.86; 0.89) | 0.13 (0.11; 0.14) | -5966.125 | 35.101 | <0.001 |
| AE | 0.93 (0.92; 0.94) |  | 0.07 (0.06; 0.08) | -5792.668 | 208.558 | <0.001 |
| E | - | - | 1.00 (1.00; 1.00) | -5117.814 | 883.412 | <0.001 |
| **Vegetables (g/d)** |  |  |  |  |  |  |
| Saturated model |  |  |  | -6215.339 |  |  |
| ACE* | 0.15 (0.12; 0.18) | 0.81 (0.78; 0.83) | 0.05 (0.04; 0.05) | -6223.647 | -8.308 | 0.002 |
| CE | - | 0.90 (0.89; 0.91) | 0.10 (0.09; 0.11) | -6176.975 | 46.672 | <0.001 |
| AE | 0.95 (0.94; 0.96) | - | 0.05 (0.04; 0.06) | -5943.109 | 280.538 | <0.001 |
| E | - | - | 1.00 (1.00; 1.00) | -5177.882 | 1045.765 | <0.001 |
| **Fruit (g/d)** |  |  |  |  |  |  |
| Saturated model |  |  |  | -6017.616 |  |  |
| ACE* | 0.10 (0.06; 0.13) | 0.82 (0.79; 0.85) | 0.09 (0.07; 0.10) | -6033.353 | -15.737 | 0.559 |
| CE | - | 0.88 (0.87; 0.89) | 0.12 (0.11; 0.13) | -6025.159 | 8.194 | <0.001 |
| AE | 0.91 (0.90; 0.92) | - | 0.09 (0.08; 0.10) | -5778.292 | 255.061 | <0.001 |
| E | - | - | 1.00 (1.00; 1.00) | -5138.175 | 895.178 | <0.001 |
| **Cereal products (g/d)** |  |  |  |  |  |  |
| Saturated model |  |  |  | -6199.339 |  |  |
| ACE* | 0.09 (0.06 ; 0.12) | 0.84 (0.81; 0.86) | 0.08 (0.06; 0.09) | -6211.289 | 11.95 | 0.041 |
| CE | - | 0.90 (0.88; 0.91) | 0.10 (0.09; 0.11) | -6201.872 | 9.417 | <0.001 |
| AE | 0.92 (0.91; 0.93) | - | 0.08 (0.07; 0.09) | -5929.622 | 281.667 | <0.001 |
| E | - | - | 1.00 (1.00; 1.00) | -5230.075 | 981.214 | <0.001 |
| **Potato (g/d)** |  |  |  |  |  |  |
| Saturated model |  |  |  | -5363.411 |  |  |
| ACE* | 0.09 (0.04; 0.15) | 0.78 (0.74; 0.82) | 0.13 (0.11; 0.15) | -5379.038 | -15.627 | 0.592 |
| CE | - | 0.84 (0.82; 0.86) | 0.15 (0.14; 0.18) | -5376.777 | 2.261 | 0.001 |
| AE | 0.88 (0.86; 0.89) | - | 0.12 (0.11; 0.14) | -5202.015 | 177.023 | <0.001 |
| E | - | - | 1.00 (1.00; 1.00) | -4707.622 | 671.416 | <0.001 |
| **Fats & oil (g/d)** |  |  |  |  |  |  |
| Saturated model |  |  |  | -5684.37 |  |  |
| ACE* | 0.05 (0.00; 0.10) | 0.84 (0.80; 0.87) | 0.12 (0.10; 0.14) | -5700.727 | -16.357 | 0.779 |
| CE | - | 0.84 (0.82; 0.86) | 0.16 (0.14; 0.18) | -5376.777 | 323.95 | 0.001 |
| AE | 0.88 (0.86; 0.89) | - | 0.12 (0.11; 0.14) | -5202.015 | 498.712 | <0.001 |
| E | - | - | 1.00 (1.00; 1.00) | -4707.622 | 993.105 | <0.001 |
| **Milk-based desserts (g/d)** |  |  |  |  |  |  |
| Saturated model |  |  |  | -975.009 |  |  |
| ACE | 0.15 (0.00; 0.30) | 0.66 (0.52; 0.77) | 0.19 (0.14; 0.26) | -986.376 | -11.367 | 0.303 |
| CE* | - | 0.76 (0.71; 0.80) | 0.24 (0.20; 0.29) | -987.39 | -1.014 | 0.054 |
| AE | 0.82 (0.77; 0.86) | - | 0.18 (0.14; 0.23) | -962.853 | 23.523 | <0.001 |
| E | - | - | 1.00 (1.00; 1.00) | -856.172 | 130.204 | <0.001 |
| **Meat & fish (g/d) ($)** |  |  |  |  |  |  |
| Saturated model |  |  |  | -7029.023 |  |  |
| ACE | 0.09 (0;0.18) | 0.86 (0.77;0.93) | 0.06 (0.03;0.1) | -7036.915 | -7.892 |  |
| CE* | - | 0.91 (0.89;0.94) | 0.09 (0.06;0.11) | -7038.922 | -2.007 | 0.08 |
| AE | 0.96 (0.94;0.98) | - | 0.04 (0.02;0.06) | -6959.954 | 76.961 | <0.001 |
| E | - | - | 1.00 (1.00; 1.00) | -6687.06 | 349.855 | <0.001 |
| **Sweet cereal products (g/d)** |  |  |  |  |  |  |
| Saturated model |  |  |  | -4721.161 |  |  |
| ACE | 0.05 (0.00; 0.10) | 0.84 (0.80; 0.87) | 0.11 (0.09; 0.14) | -4738.13 | -16.969 | 0.992 |
| CE* | - | 0.87 (0.85; 0.88) | 0.13 (0.12; 0.15) | -4739.362 | -1.232 | 0.035 |
| AE | 0.89 (0.87; 0.91) | - | 0.11 (0.10; 0.13) | -4526.581 | 211.549 | <0.001 |
| E | - | - | 1.00 (1.00; 1.00) | -4048.865 | 689.265 | <0.001 |
| **Commercial infant foods ($) (g/d)** |  |  |  |  |  |  |
| Saturated model |  |  |  | -3682.636 |  |  |
| ACE | 0.05 (0.00; 0.11) | 0.94 (0.88; 0.98) | 0.01 (0.00; 0.03) | -3692.04 | -9.404 |  |
| CE* | - | 0.97 (0.96; 0.99) | 0.03 (0.02; 0.04) | -3693.437 | -1.397 | 0.054 |
| AE | 0.99 (0.98; 1.00) | - | 0.01 (0.00; 0.02) | -3613.942 | 78.098 | <0.001 |
| E | - | - | 1.00 (1.00; 1.00) | -3409.451 | 282.589 | <0.001 |
| **Savoury snacks ($) (g/d)** |  |  |  |  |  |  |
| Saturated model |  |  |  | -4498.991 |  |  |
| ACE | 0.04 (0.00; 0.10) | 0.94 (0.88; 0.97) | 0.03 (0.01; 0.06) | -4507.116 | -8.125 |  |
| CE* | - | 0.96 (0.94; 0.97) | 0.04 (0.03; 0.06) | -4509.995 | -2.879 | 0.337 |
| AE | 0.98 (0.96; 0.99) | - | 0.02 ( 0.01; 0.04) | -4423.859 | 83.257 | <0.001 |
| E | - | - | 1.00 (1.00; 1.00) | -4203.84 | 303.276 | <0.001 |
| **Added sugars & confectionery ($) (g/d)** |  |  |  |  |  |  |
| Saturated model |  |  |  | -5152.39 |  |  |
| ACE | 0.05 (0.00; 0.13) | 0.91 ( 0.84; 0.96) | 0.04 (0.02; 0.08) | -5157.881 | -5.491 |  |
| CE* | - | 0.94 (0.92; 0.96) | 0.06 (0.04; 0.08) | -5160.763 | -2.882 | 0.311 |
| AE | 0.98 (0.95; 0.99) | - | 0.02 (0.01; 0.05) | -5078.926 | 78.955 | <0.001 |
| E | - | - | 1.00 (1.00; 1.00) | -4863.978 | 293.903 | <0.001 |
| **Egg (g/d) ($)** |  |  |  |  |  |  |
| Saturated Model |  |  |  | -2770.787 |  |  |
| ACE | 0.06 (0;0.15) | 0.91 (0.82; 0.97) | 0.03 (0.01; 0.07) | -2776.155 | -5.368 |  |
| CE* | - | 0.95 (0.92; 0.97) | 0.05 (0.03; 0.08) | -2778.691 | -2.536 | 0.273 |
| AE | 0 (0.98;0.95) | - | 0.02 (0.01; 0.05) | -2729.61 | 46.545 | <0.001 |
| E | - | - | 1.00 (1.00; 1.00) | -2586.754 | 189.401 | <0.001 |
| ($) dichotomised variable; * best fitting model | | | | | | |
